# Bilateral adrenal metastasis of renal cell carcinoma 4 years after radical nephrectomy: A case report and review of literature

**DOI:** 10.1097/MD.0000000000026838

**Published:** 2021-08-06

**Authors:** Yingjie Li, Zhigang Ji, Dong Wang, Yi Xie

**Affiliations:** Department of Urology, Peking Union Medical College Hospital, Chinese Academy of Medical Science, Beijing, China.

**Keywords:** adrenal metastasis, bilateral adrenalectomy, case report, laparoscopy, radical nephrectomy, renal cell carcinoma

## Abstract

**Rationale:**

Renal cell carcinoma (RCC) almost metastasizes to every organ, the possibility of adrenal metastasis is relatively low in patients that have undergone radical nephrectomy, only a few cases of bilateral adrenal metastasis are reported on literature. Although surgical treatment of metastases from RCC is preferred and contributes to the rate of survival, it is considered challenging to manage such cases due to the rarity of bilateral metastasis to the adrenal glands.

**Patient concerns:**

A 64-year-old Manchus female presented with an incidental ultrasonic finding of a left adrenal mass 4 years after radical nephrectomy for left renal cell carcinoma.

**Diagnosis:**

Abdominal contrast enhanced CT scan revealed bilateral adrenal masses, suggesting metastatic lesion. Examinations indicated neither local recurrence nor distant metastasis anywhere have been detected by whole body Positron Emission Tomography/Computed Tomography (PET/CT) scan except high radioactive uptake in bilateral adrenal glands.

**Interventions:**

Metachronous bilateral adrenalectomy was taken and laparoscopic right adrenalectomy was first performed. She was discharged home on third postoperative day. Pathological examination revealed metastatic renal cell carcinoma. Two months later she was performed laparoscopic left adrenalectomy.

**Outcomes:**

The patient healed without obvious complications and no tumor recurrence.

**Lessons:**

Bilateral metastatic adrenal recurrence from RCC is very rare. Early diagnosis of adrenal metastasis is challenging because they are usually silent both anatomically and functionally. Surgical intervention is a wise option for these patients that may improve survival, and metachronous bilateral adrenalectomy is proved to be safe and effective.

## 1 Introduction

Among all types of cancer worldwide, renal cell carcinoma (RCC) account for 2–3%, and is known for metastasis to almost every organ. The most common sites are lung (50–60%), liver (30–40), bone (30–40%) and brain (5%). However, the possibility of adrenal metastasis is relatively low. The incidence of solitary ipsilateral and contralateral adrenal metastasis was 3–5% and 0.7% respectively in patients who had undergone radical nephrectomy,^[1]^ only a few cases of bilateral adrenal metastasis are reported in literature. Although surgical treatment of metastases from RCC is preferred and contributes to the rate of survival,^[2]^ it is considered challenging to manage such cases due to the rarity of bilateral metastasis to adrenal glands. Here we present a patient with bilateral adrenal metastases 4 years after left radical nephrectomy who underwent metachronous bilateral adrenalectomy in our hospital.

## 2 Case presentation

**Figure 1:**
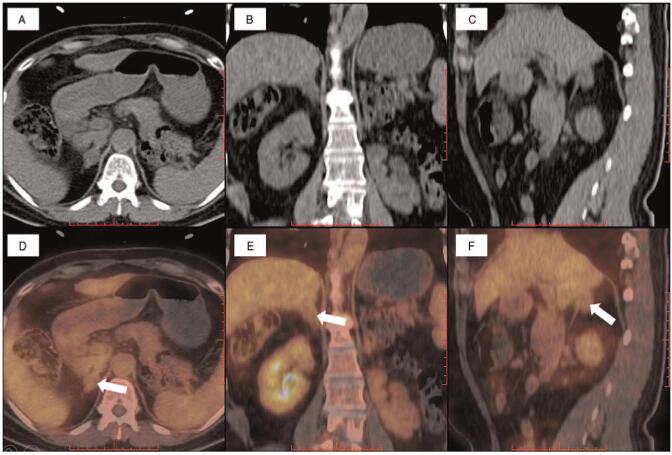
PET/CT scan showed high radio uptake mass on the right adrenal gland (white arrows). (A&D) Axial PET/CT images: CT image and fusion image. (B&E) Coronal PET/CT images: CT image and fusion image. (C&F) Sagittal PET/CT images: CT image and fusion image.

**Figure 2:**
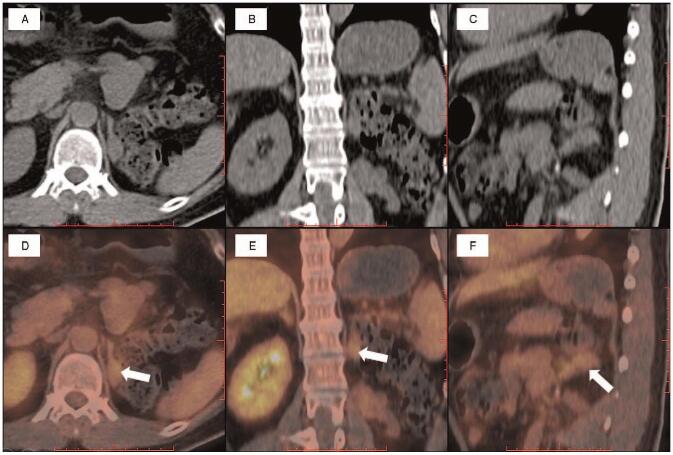
PET/CT scan showed high radio uptake mass on left adrenal gland (white arrows). (A&D) Axial PET/CT images: CT image and fusion image. (B&E) Coronal PET/CT images: CT image and fusion image. (C&F) Sagittal PET/CT images: CT image and fusion image.

A 64 -year-old Manchus female presented with incidental ultrasonic evidence of left upper pole renal mass 4 years ago. Further evaluation with abdominal CT urography revealed a mass in the left kidney with uneven enhancement, and multiple masses in bilateral kidneys with low density and no enhancement signal. The right renal mass was identified as right renal cyst through laparoscopic approach while the left radical nephrectomy was performed sparring the left adrenal gland. Pathology specimen analysis suggested renal cell carcinoma, Grade II Fuhrman nuclear characteristics, confined to the capsule, neither pelvicalyceal nor vascular invasion was found (pT1bN0M0). Postoperatively she did not receive immunotherapy or chemotherapy, the patient was on regular follow up and no local or metastatic recurrence was found until March 2018, an incidental mass was found in the left adrenal gland during a checkup visit for the status of her right solitary kidney. Abdominal and pelvic CT scan was done, revealing a nodular mass on the left adrenal gland. Due to the solitary finding and no clinical symptom was found, palliative observation was recommended. Three months later bilateral adrenal masses were found and subsequent checkup revealed enlargement of bilateral adrenal masses. Whole body Positron Emission Tomography/Computed Tomography (PET/CT) scan with fluorodeoxyglucose was performed, suggesting bilateral adrenal nodules which had high radioactive uptakes. The right one measures 2.0 × 2.4 × 2.6 cm approximately, with the SUV is 3.0 (Fig. 1); The Size of left adrenal mass is about 1.6 × 2.9 × 2.7 cm, while the SUV is 2.8 (Fig. 2). The result indicated the probability of metastasis from renal cell carcinoma. Except this, neither local nor metastatic recurrence was observed in any system.

**Figure 3:**
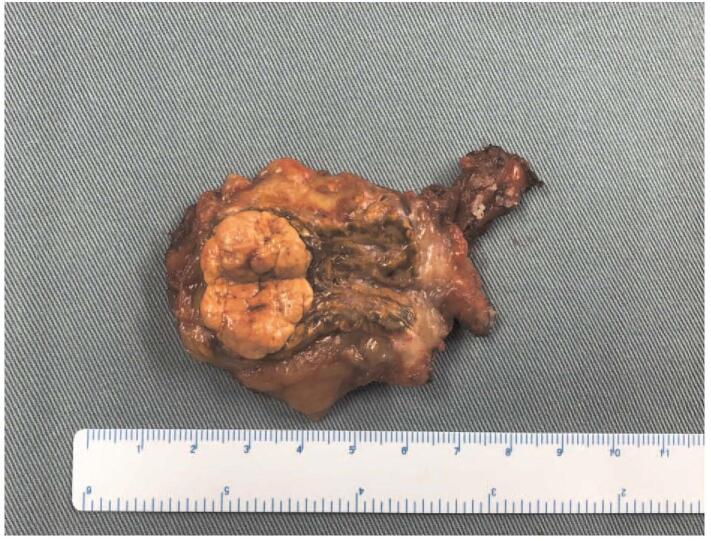
Left adrenal mass was extracted.

**Figure 4:**
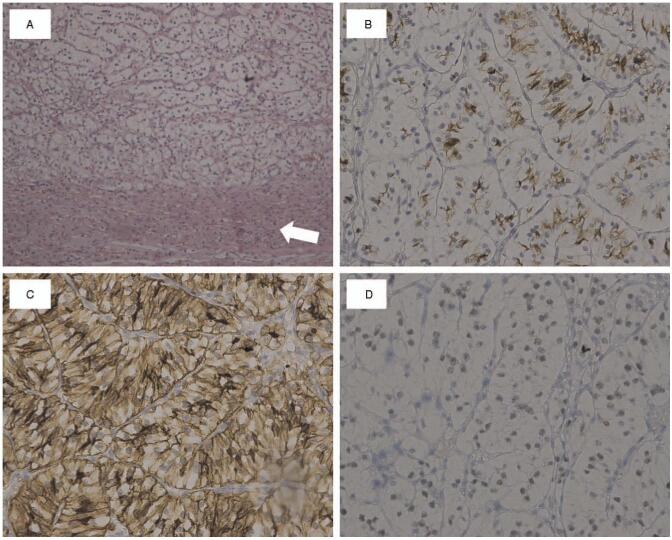
Pathological and immunohistochemical examination of left adrenal mass showed metastatic renal cell carcinoma. (A) Photomicrograph showing renal cell carcinoma replacing normal adrenal cortex (white arrow) (hematoxylin and eosin; magnification 100 × ). The neoplastic cells are positive for (B) RCC (magnification 200 ×), (C) CD10 (magnification 200 ×) and (D) PAX-8 (magnification 200 ×).

The patient was admitted for further investigation, thorough hematological, biochemical and hormonal examinations were performed, all results were within normal range. Laboratory examinations showed the adrenal masses to be nonfunctional. To clarify diagnosis, managed the deterioration and avoided the development of iatrogenic Addison's disease, metachronous bilateral adrenalectomy was taken for the current case. On July 2019, under general anesthesia, in left lateral position through laparoscopy, right adrenalectomy was first done considering that lesion on the right side seems more severe. No any perioperative complications were recorded and she was discharged home on third post-operative day. Pathological examination revealed morphological and immunohistochemical findings in line with metastatic renal cell carcinoma, including positive staining for AE1/AE3 vimentin and CD10, partial positive for RCC, PAX-8, and negative staining for inhibin, CgA, and CA9, the index of Ki-67 is 5%. With the evidence of pathological result and efficacy of surgical resection, she was performed left side adenectomy through laparoscopy two months later (Fig. 3). Pathological examination revealed morphological and immunohistochemical findings in line with metastatic renal cell carcinoma, including positive staining for RCC, PAX-8, vimentin and CD10, partial positive for AE1/AE3, and negative staining for Ck7, Syn, α-inhibin, CgA, Melan-A and GATA3, the index of Ki-67 is 20% (Fig. 4). She was discharged 3 days after surgery and undergoes steroid replacement therapy right now with no sign of adrenal insufficiency. New metastases or recurrence were not observed at the 6^th^ month of follow-up.

## 3 Discussion and conclusion

Metastasis can be found synchronously with RCC or many years after nephrectomy.^[3]^ In general, adrenal involvement from primary tumor of renal is rare, and only a few literatures reported bilateral adrenal metastasis in patients with RCC. Based on previous studies, synchronous ipsilateral adrenalectomy with radical surgery of RCC is not routinely recommended as the risk of ipsilateral metastasis is low except selected patients that have a large, advanced T-stage and upper pole tumor.^[4,5]^

However, the diagnosis of adrenal metastasis is challenging. A study suggested that a number of adrenal metastasis in patients treated with radical nephrectomy are considered to be underdiagnosed because metastases are usually asymptomatic both functionally and anatomically.^[1]^ Imaging methods can help locate the mass but fail to determine whether an adrenal tumor is a primary adrenocortical carcinoma, benign adenoma or metastasis from the renal tumor. Therefore, hormonal examination is recommended for patients after radical nephrectomy who have detected adrenal lesions by radiological examination. In the case of normal hormone level, PET/CT scan may assist clinical diagnosis. Augmentative radiological uptake ratio with normal hormone examination results suggests metastatic lesion.^[6]^ The present case follows this protocol and confirmed the metastasis diagnosis.

In most previously published cases, adrenal metastases occurred simultaneously with RCC, therefore the available information on metachronous metastasis is incomplete and relevant therapeutics experience is limited. Nevertheless, with the advance of surgical technology, adrenalectomy with the aim of prolonging survival has been regarded as the standard of care in patients with adrenal metastasis.^[7]^ Laparoscopic adrenalectomy has advantages such as rapid recovery, less pain, better cosmetic results, lower percentage of incision hernia, and rapid return to possible systemic therapy for metastatic diseases.^[8]^ Therefore, laparoscopic adrenalectomy is the preferred treatment for adrenal gland metastasis in RCC patients. To date, various surgical modalities have been implemented to improve the survival rate. Specific surgical procedure depends on the condition of patients (Table 1). In a case reported by Akbar,^[8]^ bilateral laparoscopic adrenalectomy for adrenal metastasis is considered to be a feasible approach, while another study by Tanica^[9]^ preferred unilateral adrenal removal followed by immunotherapy in case of development of iatrogenic Addison's disease. Mohammad^[10]^ also performed the operation of unilateral adrenalectomy with the other side adrenal mass preserving due to the poor general condition and to prevent adrenal insufficiency during surgery. In light of information obtained and overall consideration of the patient, to manage the malignance progression, we took the strategy of metachronous bilateral adrenalectomy followed by steroid replacement therapy that has not been adopted before. During her first admission, contralateral adrenalectomy was performed to clarify the diagnosis and avoid stressful adrenal insufficiency caused by the abrupt elimination of both adrenal glands. Moreover, under the condition that pathological examination showed metastases from renal cell carcinoma and no adverse event was observed after operation, ipsilateral adrenalectomy was then implemented in her followed admission. Based on our experience, the protocol of metachronous bilateral adrenalectomy is a feasible and effective approach, but extensive experience is required in laparoscopic surgery modality to treat patients with bilateral adrenal metastasis from RCC.

**Table 1.** Renal cell carcinoma with synchronous bilateral adrenal metastases.

				Size (cm)					
Reference	Age/sex	Primary site	Discovery time	Primary	Rt-ad	Lt-ad	Surgery	Post-op therapy	Outcome
1. Tsuboniwa et al^[11]^	72/M	L	Syn	10	2	2	Lt Rad NxBil Adnx	interferon therapy	Six months alive
2. Koutalellis et al^[12]^	58/F	R	Syn	6	2	6	Rt Rad NxBil Adnx	adjuvant chemotherapy	Six months Alive
3. Moslemi et al ^[10]^	72/M	L	Syn	14	5	5	Lt Rad NxLt Adnx	immunotherapy	One year alive
4. Ozturk et al^[13]^	61/M	L	Met (2y after Sur)	-	4	5	Lt AdnxRt Par Adnx	–	Six months alive
5. Ozturk et al ^[14]^	50/M	R	Syn	8	2	7	Rt Rad NxRt AdnxLt Par Adnx	–	–
6. Gokcen et al ^[15]^	54/M	L	Syn	6	7	14	Lt Rad NxBil Adnx	–	–
7. Nouralizadeh et al ^[8]^	64/F	L	Met (7y after Sur)	–	4	4	Bil Adnx	–	–
8. Pandey et al ^[9]^	60F	Bil	Syn	R2L3	–	–	Rt Par NxLt Par NxRt Adnx	immunotherapy	Six months alive

In conclusion, bilateral adrenal metastatic recurrence of RCC is extremely rare event. Radiology methods combined with hormone examination can help in diagnosis of adrenal metastasis. Laparoscopic surgical removal of adrenal masses is a wise option that may improve survival. The strategy metachronous bilateral adrenalectomy is proved to be effective with no complications in patients with bilateral involvement after radical nephrectomy. Yet the therapeutic modalities still need to be investigated in further studies and more cases. Among the literature reporting bilateral metachronous adrenal metastases of operated renal cell carcinoma, none of them mentioned continued therapy after operation of primary tumor. Several factors may contribute to adrenal metastasis from renal cell carcinoma, such as large tumor, upper location and advanced pathology finding. Systemic therapy is highly recommended for patients with risk factors.

## Author contributions

**Conceptualization:** Yi Xie.

**Supervision:** Zhigang Ji, Dong Wang, Yi Xie.

**Visualization:** Yingjie Li.

**Writing – original draft:** Yingjie Li.

**Writing – review & editing:** Yingjie Li, Zhigang Ji, Dong Wang, Yi Xie.
